# Identification of a Four-Gene Metabolic Signature to Evaluate the Prognosis of Colon Adenocarcinoma Patients

**DOI:** 10.3389/fpubh.2022.860381

**Published:** 2022-04-07

**Authors:** Yang Zheng, Rilige Wu, Ximo Wang, Chengliang Yin

**Affiliations:** ^1^Graduate School, Tianjin Medical University, Tianjin, China; ^2^Tianjin Key Laboratory of Acute Abdomen Disease Associated Organ Injury and ITCWM Repair, Institute of Integrative Medicine for Acute Abdominal Diseases, Tianjin Nankai Hospital, Tianjin, China; ^3^College of Science, Beijing University of Posts and Telecommunications, Beijing, China; ^4^Tianjin Haihe Hospital, Tianjin, China; ^5^Faculty of Medicine, Macau University of Science and Technology, Macau, China

**Keywords:** COAD, metabolic, LASSO, Cox, signature, prognostic prediction

## Abstract

**Background:**

Colon adenocarcinoma (COAD) is a highly heterogeneous disease, thus making prognostic predictions uniquely challenging. Metabolic reprogramming is emerging as a novel cancer hallmark that may serve as the basis for more effective prognosis strategies.

**Methods:**

The mRNA expression profiles and relevant clinical information of COAD patients were downloaded from public resources. The least absolute shrinkage and selection operator (LASSO) Cox regression model was exploited to establish a prognostic model, which was performed to gain risk scores for multiple genes in The Cancer Genome Atlas (TCGA) COAD patients and validated in GSE39582 cohort. A forest plot and nomogram were constructed to visualize the data. The clinical nomogram was calibrated using a calibration curve coupled with decision curve analysis (DCA). The association between the model genes' expression and six types of infiltrating immunocytes was evaluated. Apoptosis, cell cycle assays and cell transfection experiments were performed.

**Results:**

Univariate Cox regression analysis results indicated that ten differentially expressed genes (DEGs) were related with disease-free survival (DFS) (*P*-value< 0.01). A four-gene signature was developed to classify patients into high- and low-risk groups. And patients with high-risk exhibited obviously lower DFS in the training and validation cohorts (*P* < 0.05). The risk score was an independent parameter of the multivariate Cox regression analyses of DFS in the training cohort (HR > 1, *P*-value< 0.001). The same findings for overall survival (OS) were obtained GO enrichment analysis revealed several metabolic pathways with significant DEGs enrichment, G1/S transition of mitotic cell cycle, CD8+ T-cells and B-cells may be significantly associated with COAD in DFS and OS. These findings demonstrate that si-FUT1 inhibited cell migration and facilitated apoptosis in COAD.

**Conclusion:**

This research reveals that a novel metabolic gene signature could be used to evaluate the prognosis of COAD, and targeting metabolic pathways may serve as a therapeutic alternative.

## Introduction

Metabolic reprogramming has been recognized as one of the distinguishing feature of tumor cells recently (1). Cancer metabolism is a major contributor to tumor initiation, growth, and metastasis in colorectal cancer (2).

Colon adenocarcinoma (COAD) is a primary intestinal aggressive malignancy (3). It is the third most prevalent cancer (10.0%) and is ranked second (9.4%) in cancer-related mortality worldwide (4). Further, this disease is genetically heterogeneous whose prognosis is uniquely challenging. China, with the most population in the world, is also ranked first in terms of new cases of cancer and cancer-related deaths in the world (5, 6). In China, more than 555,000 new colon and rectum cancer cases are projected to be discovered annually (4, 6). The world's cancer burden including COAD continues to increase and malignancy is quickly becoming the foremost cause of human death in this century (7). Therefore, predicting the prognosis of COAD patients and identifying new therapeutic strategies for COAD are critical.

According to previous reports, metabolism serves a vital role in the progress of COAD and some key genes such as OMA1 mediate metabolism reprogramming under hypoxia, thus facilitating colorectal cancer development by promoting the Warburg effect (8). And LINRIS stabilizes IGF2BP2 and facilitates aerobic glycolysis in COAD (9). However, the link between metabolic-related genes and the prognosis of COAD patients remains largely uncharacterized. Metabolism and expression of specific genes affect the occurrence of metabolism, making it a therapeutic potential target for the management of malignant tumor (10). Thus, it is important to gain further understanding of the underlying metabolic mechanisms of COAD tumorigenesis and progression. Further, it is needed urgently to identify new prognostic-related markers which could become therapeutic potential targets in COAD (11, 12).

First, we acquired expression profiles of mRNA and the relevant clinical features of COAD patients from open data resources in our research. We then established a multigene prognostic characteristic of COAD by metabolic-related differentially expressed genes (DEGs) in a Gene Expression Omnibus (GEO) cohort. Finally, functional enrichment analysis was performed to explain the potential mechanisms involved, after which a prognostic model was developed based on several metabolic-related genes. We consider that this prognostic model with the strong predictive power of COAD will improve prognosis risk assessment of patients with COAD and help to develop a more accurate evaluation of their clinical management.

## Materials and Methods

### Publicly Available mRNA Data and Metastasis Gene Sets

An RNA-sequencing (RNA-seq) dataset and relevant clinical data of COAD patients were acquired from The Cancer Genome Atlas colon adenocarcinoma dataset (TCGA-COAD) and GEO website (GSE25071) to compare human colorectal tumors and normal colorectal tissues. The normalization of gene expression profiles was performed by the scale method provided in the “limma” package in R. We then identified DEGs though the comparison of patients with COAD with recurrent and normal humans in the GEO cohort (GSE21510 and GSE32323). A total of 566 COAD patients from the training set of the TCGA-COAD cohort and 573 COAD patients from the GSE39582 cohort used for the validation set were eventually enrolled. A list of metabolic-related genes was then gathered from the Gene Set Enrichment Analysis (GSEA, v3.0, http://software.broadinstitute.org/gsea/index.jsp) website (13) and is exhibited in Supplementary Table 1.

### Building and Verification of a Prognostic Model of Metabolic-Related Genes

DEGs with log (|fold change|) >1 and a false discovery rate (FDR) < 0.05 between tumor tissues and adjacent non-cancerous tissues in the TCGA-COAD and GSE25071 datasets were identified by the “limma” package in R. Differences between the recurrence and normal human GEO cohorts (GSE21510 and GSE32323, respectively) were also evaluated. Univariate Cox analysis of disease-free survival (DFS) and overall survival (OS) in DEGs were carried out to identify the metabolic-related genes with prognostic significance. We utilized the log-rank test to adjust the *p*-value.

To reduce the number of genes, we used the least absolute shrinkage and selection operator (LASSO) Cox regression analysis to screen genes for DFS and OS, respectively. The input of the LASSO Cox regression analysis were the above-mentioned screened genes. The most vital value in the LASSO Cox regression analysis was λ. Using different λ values, LASSO Cox regression analysis can be utilized to screen for different genes. We used cross-validation to select the optimal λ for DFS and OS. With these λ values, the genes associated with DFS and OS were identified via LASSO Cox regression analysis, respectively. These genes were then subjected to multivariate Cox regression analysis to determine their coefficients for the prognostic models of DFS and OS, respectively. The prognostic models were represented as a risk score, which was expressed as follows:


$$\begin{array}{l} \mathrm{Risk}\;\mathrm{Score}\;=\overset{N}{\underset{i=1}{\sum }}\mathrm{Expi}\times \mathrm{Coefi}\; \end{array}$$

A total of 222 patients obtained from the TCGA-COAD cohort with a DFS survival status and survival time were classified into low- and high-risk groups by the best cut-off point determined by the surv_cutpoint function in the “Survminer” package in R. We then plotted the survival curves for the risk score via the “survfit” function with the “survival” R package. We did the same for the 214 patients included in the TCGA-COAD data set who had the stages (stages I, II, and III) information of all those 222 patients, 51 patients for stage I, 147 patients for stage I and II, and 67 patients for stage III. To appraise the predictive power of the risk score, we plotted the receiver operating characteristic (ROC) and displayed the 1-, 3- and 5-year projections as function of survival ROC in the “survivalROC” R package.

### Validation of the Prognostic Model as an Independent Clinical Factor

To explore the correlation of the established prognostic model with the clinical information, we carried out univariate cox regression analyses to generate risk scores and consider other clinical features including stage, sex, and age in 214 DFS patients included in the TCGA-COAD data set. Risk scores and other clinical factors were deemed statistically relevant at a *p*-value threshold of <0.05. Association between risk score levels and other clinical factors with DFS and OS were investigated by multivariate Cox proportional hazard models. The results of the multivariate Cox regression model were visualized in a combined forest plot and the same procedure was also performed in TCGA OS patients and the validation data set.

### Verification of the Nomogram

A nomogram was created to forecast the DFS of patients at 1-, 3-, and 5-year survival probabilities to help doctors understand and apply the model. Then, we performed ROC analysis to gain the area under the curve (AUC) and verify the predictive effect of the model. The same procedure was performed in TCGA OS patients and the validation data set. The calibration curve of the nomogram was drawn to validate the nomogram's predictive value. The calibration curve was produced by using the calibration function in the “rms” package in R.

The decision curve analysis (DCA) results were plotted to quantify and assess the clinical value of the nomogram (14). DCA can be performed to obtain the clinical net benefit of the nomogram compared with all or none of the strategies (15).

### Functional Enrichment Analysis and Cell Immune Infiltration

A comprehensive knowledge-base update to the sixth version of the original web-based programs is provided by the Database for Annotation, Visualization and Integrated Discovery (DAVID, v6.8, https://david.ncifcrf.gov/). With the comprehensive set of function annotation tools provided by DAVID, researchers can examine the biological meanings of a large set of genes. The Gene Ontology resource (GO; http://geneontology.org) provides structured, computable knowledge regarding the functions of genes and gene products (16). The knowledge of GO resource is both human-readable and machine-readable, and is a foundation for computational analysis of large-scale molecular biology and genetics experiments in biomedical research. DAVID was used to conduct GO analysis based on genes in a univariate Cox analysis of OS and DFS.

Tumor Immune Estimation Resource (TIMER; https://cistrome.shinyapps.io/timer/) is a web interactive platform and is used to analyse tumor-infiltrating immunocytes systematically. We utilized TIMER to study the correlation of genes in the risk score model and the signatures of tumor-infiltrating immunocytes in COAD. The “Gene” module was carried out to investigate the relevance between the risk score model expression and immunocyte infiltration levels specific to each gene (B-cells, neutrophils, macrophages, dendritic cells, CD4+ and CD8+ T-cells) with the TCGA database. We applied TIMER to explore the relationship between the gene expression in the risk score model and the marker gene sets of different immunocytes with the “Correlation” module. The relationship between the gene expression in the risk score model and the tumor-infiltrating immune cells were assessed by purity-correlated partial Spearman's correlation and statistical significance (17).

### External Validation Using Online Databases

Hub genes in LASSO Cox were surveyed from the following online databases: (1) Oncomine database analysis. Oncomine database (https://www.oncomine.org/resource/main.html) is a tumor microarray database and online data analysis tool that collects many “multi-arrays” (18). This tool was used to determine gene expression signatures. Gene expression levels in various types of cancers were identified using the Oncomine database at a *p*-value threshold of 0.001 and a fold change threshold of 2 using gene ranking. The following conditions were used to acquire the mRNA expression level in tumor tissue compared with normal tissues: *P* < 1E-4, fold change > 2, and top gene rank 10% (19). (2) cBioPortal analysis. The cBioPortal for Cancer Genomics (http://cbioportal.org) was constructed specifically to decrease the difficulty of obtaining complex data sets and promote the translation of genomic data into novel biological knowledge, treatments, and clinical trials (20). This platform encourages the study of multidimensional tumor genomics data by supporting visualization and estimating across genes, samples, and data types. The resource allows researchers to visualize the patterns of gene alterations across samples in one tumor research, to compare the frequencies of gene alterations across multiple tumor researches, and to aggregate alterations of all related genomes in an single cancer sample. cBioPortal encompasses multiple genomic data types such as somatic mutations, DNA copy number alterations (CNAs), mRNA and microRNA (miRNA) expression, DNA methylation, protein abundance, and phosphoprotein abundance (21). (3) PrognoScan database Analysis. The PrognoScan database (http://www.abren.net/PrognoScan/) was utilized to explore the association between gene expression and survival across various types of cancers. PrognoScan was utilized to dissect the correlations between gene expression and prognosis indicators (e.g., OS and DFS) across a large number of open tumor microarray datasets. The threshold of Cox *p*-value was adjusted to <0.05 (22). (4) GEPIA database Analysis. The Gene Expression Profiling Interactive Analysis (GEPIA, http://gepia.cancer-pku.cn/) database is a developed interactive web server for analyzing the RNA sequencing expression data of tumor and normal samples from the TCGA and the GTEx projects, using a standard processing pipeline. GEPIA provides customizable functions such as tumor/normal differential expression analysis, profiling according to cancer types or pathological stages, patient survival analysis, similar gene detection, correlation analysis and dimensionality reduction analysis (23).

### Apoptosis and Cycle Assay

The analysis of the previous sections indicated that FUT1 was an aberrantly expressed gene in COAD tumors and therefore high FUT1 expression could be used as a predictor of adverse outcomes. So we examined the function of FUT1 in cell apoptosis and cell cycle and performed a flow cytometry assay. We cultured HCT-116 cells for 24 h with FUT1 and then harvested the cells. Next, Annexin V and propidium iodide (PI) were used in accordance with the manufacturer's recommendation (Beijing Solarbio, China). The apoptotic rate and cell cycle were detected using a NovoCyte flow cytometer (ACEA, Biosciences, USA) and analyzed using the NovoExpress software (ACEA, Biosciences, USA).

### Cell Transfection

Cell transfection was also performed to further verify the effect of FUT1. HCT-116 cells were gained from the American Type Culture Collection. The cells were kept in Dulbecco's modified Eagle's medium (DMEM) (Gibco, USA), supplemented with 10% fetal bovine serum (FBS) (Gibco, USA), 100 μg/mL streptomycin (Gibco, USA) and 100 U/mL penicillin (Gibco, USA) in a humidified incubator with 37°C and 5% CO_2_. FUT1 was silenced in HCT-116 cells by transfection with FUT1 short interfering RNA (si-FUT1). si-FUT1 and the siRNA negative control (si-NC) were purchased from Tsingke Biotech (Beijing, China). Lipofectamine 3000 (Invitrogen, Carlsbad, CA, USA) was used to carried out the transfection trials according to the manufacturer's instructions.

## Results

### Patient Selection

A total of 566 COAD patients were selected as the training set from TCGA and 573 COAD patients were selected from the GSE39582 cohort as the validation set. The specific clinical features of the patients were listed in Table 1. A flow chart of our research was illustrated in Supplementary Figure 1.

**Table 1:** Clinical characteristics of colorectal patients.

**Characteristics**	**TCGA (*N* = 566)**	**GSE39582 (*N* = 573)**
AGE, Median (Q1,Q3)	67 (57, 75)	68 (59, 76)
**Gender**, ***n*** **(%)**		
Female	264 (47%)	256 (45%)
Male	302 (53%)	317 (55%)
**ANNARBOR.Stage**, ***n*** **(%)**		
NA	12 (2%)	–
0	–	4 (1%)
I	101 (18%)	37 (6%)
II	207 (37%)	265 (46%)
III	165 (29%)	208 (36%)
IV	81 (14%)	59 (10%)

### Identifying of Prognostic Metabolic-Related DEGs in the TCGA Cohort

A total of 153 overlapping DEGs were identified between (1) tumor tissues and non-tumor adjacent tissues in TCGA and GSE20571, and (2) recurrent and normal human tissues in the GEO cohort (GSE21510 and GSE32323). A total of 62 genes were identified by matching 153 DEGs with metabolic-related genes, 21 of which were correlated with OS and 10 were correlated with DFS in the univariate Cox regression analysis (*P* < 0.01) in the training set (Tables 2, 3).

**Table 2:** Disease-free survival associated gene list in COAD from the training set (TCGA).

**No**.	**Gene**	**HR**	***P*** **-value**
1	CCND1	0.238875	0.000367
2	CXCL1	2.968093	0.007837
3	CXCL10	3.176181	0.002312
4	CXCL3	2.856345	0.005621
5	EDAR	0.190756	3.90E-05
6	FUT1	0.358673	0.008883
7	INHBA	0.31451	0.001944
8	MMP1	4.725989	0.000239
9	MYC	2.978401	0.00377
10	PPAT	2.960687	0.006917

**Table 3:** Overall survival associated gene list in COAD from the training set (TCGA).

**No**.	**Gene**	**HR**	***P*** **-value**
1	CCNB1	2.639522	0.000693
2	CCND1	0.535604	0.005232
3	CDC6	1.75829	0.002691
4	CDH3	1.924981	0.008129
5	CXCL1	1.743645	0.005178
6	CXCL11	1.937543	0.001367
7	CXCL3	1.765604	0.00236
8	FUT1	0.583245	0.007906
9	GPD1L	1.832985	0.004354
10	MAD2L1	1.977956	0.000292
11	MMP1	2.060513	0.000426
12	MYC	1.670215	0.009585
13	NME1	2.070147	0.001144
14	PGM1	2.00308	0.001948
15	PPAT	2.180598	0.001574
16	PPIL1	1.822861	0.001349
17	PTTG1	1.746729	0.003743
18	RIPK2	2.426172	0.000253
19	RRM2	1.896863	0.001508
20	SPP1	0.519043	0.000739
21	TKT	1.957591	0.000346

### Establishment of a Prognostic Model in the TCGA Cohort

**Figure 1:**
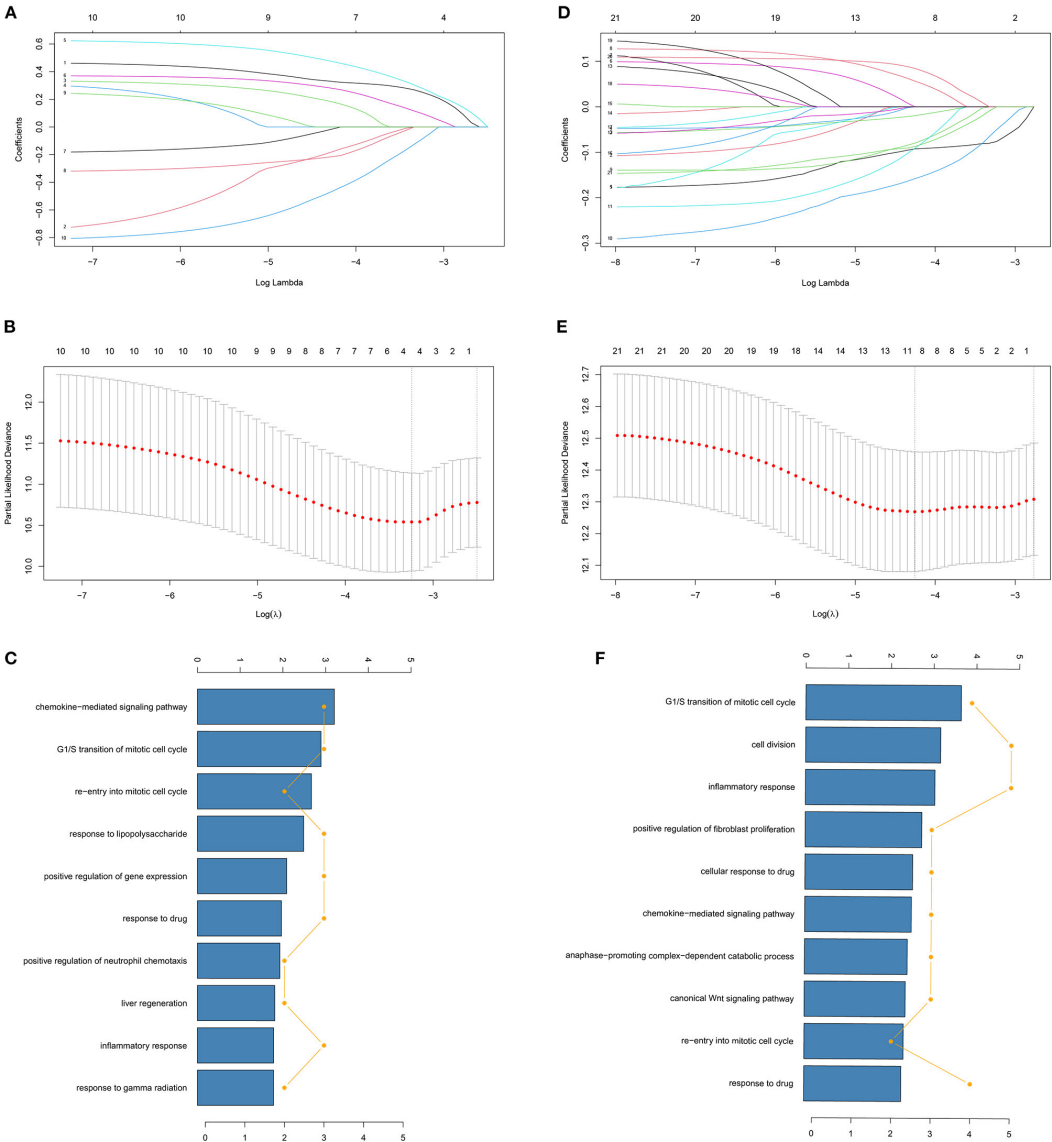
Identification of genes screened via LASSO cox regression analysis. **(A,D)** Coefficients of different genes screened via LASSO cox regression analysis with different λ in DFS and OS. The two dashed lines represent lambda.min and lambda.1se and lambda.min was selected for both DFS and OS. **(B,E)** Partial likelihood deviance of cross-validation with different λ in DFS and OS. **(C,F)** GO analysis of identified genes of LASSO cox regression analysis in DFS and OS.

We used LASSO Cox regression analysis to both the 10 DFS genes and 21 OS genes to develop prognostic models with the expression profile of the above-mentioned genes. LASSO Cox regression analysis was used to screen four genes (CCND1, EDAR, FUT1, and PPAT) in DFS and eight genes (CCNB1, CDC6, FUT1, GPD1L, MAD2L1, MMP1, SPP1, and TKT) in OS, which acted as the input for multivariate Cox regression analysis. We obtained the coefficients of the prognostic models of both DFS and OS using multivariate Cox regression analysis (Figures 1A,B,D,E). Finally, prognostic models were displayed in the form of a risk score as follows:


$$\begin{array}{l} \mathrm{Risk}\;\mathrm{score}\;\mathrm{of}\;\mathrm{DFS}\;=\text{​}0.411228099154717\;\times \;\mathrm{CCND}1\;\\ \;+\;0.547206032433896\;\times \;\mathrm{EDAR}\;\\ \;+\;0.302185127495718\;\times \;\mathrm{FUT}1\;\\ \;-\;0.583673054198275\;\times \;\mathrm{PPAT}\;\\ \;\mathrm{Risk}\;\mathrm{score}\;\mathrm{of}\;\mathrm{OS}\;=\;-0.0985300797882395\;\times \;\mathrm{CCNB}1\;\\ \;-\;0.0566241392969015\;\times \;\mathrm{CDC}6\;\\ \;+\;0.111288163590287\;\times \;\mathrm{FUT}1\;\\ \;-\;0.144434028275748\;\times \;\mathrm{GPD}1L\;\\ \;-\;0.21174022083491\;\times \;\mathrm{MAD}2L1\;\\ \;-\;0.231299794085908\;\times \;\mathrm{MMP}1\;\\ \;+\;0.129051446614179\;\times \;\mathrm{SPP}1\;\\ \;-\;0.140224178453846\;\times \;\mathrm{TKT} \end{array}$$

GO term analyses were carried out to investigate the potential biological meanings of the 21 identified DFS genes and 10 OS genes. As depicted in Figures 1C,F, GO annotation revealed top categories that were positively correlated with metabolism in DFS such as chemokine-mediated signaling pathway, G1/S transition of mitotic cell cycle, and re-entry into mitotic cell cycle, among others. Other pathways were identified for OS including G1/S transition of mitotic cell cycle, cell division, and inflammatory response. These results indicate that GO enrichment is critically important in COAD patients and is strongly associated with metabolism, especially in the G1/S transition of the mitotic cell cycle.

The relevance between the gene expression in the risk score DFS model and six types of infiltrating immunocytes (B-cells, neutrophils, macrophages, and dendritic cells, CD4+ and CD8+ T-cells) was examined further. The DFS analysis indicated that 3/4 of the expression levels in the risk score model were obviously related with the infiltrating of B-cells, CD8+ T-cells, and macrophages (Supplementary Figure 2). The EDAR coefficient in the risk score of DFS was positive and EDAR expression was positively associated with the levels of B-cell and CD8+ T-cell infiltrating in COAD (Supplementary Figure 2B). Our analysis indicates that CD8+ T-cells and B-cells may be obviously associated with COAD.

We also investigated the correlation between the gene expression in the risk score OS model expression and 6 types of infiltrating immunocytes. The OS analysis indicated that the genes in the risk score OS model were associated with CD8+ T and B-cell infiltrating. The coefficients of FUT1 and SPP1 in the risk score of OS were positive, and FUT1 expression was critically correlated with the levels of B cell infiltrating in COAD (Supplementary Figure 3C). SPP1 expression was critically associated with the infiltrating degrees of CD8+ T-cells in COAD (Supplementary Figure 3F). Above all, we considered that CD8+ T-cells and B-cells may be obviously correlated with COAD in both DFS and OS.

### Independent Prognostic Role of the Prognostic Model

**Figure 2:**
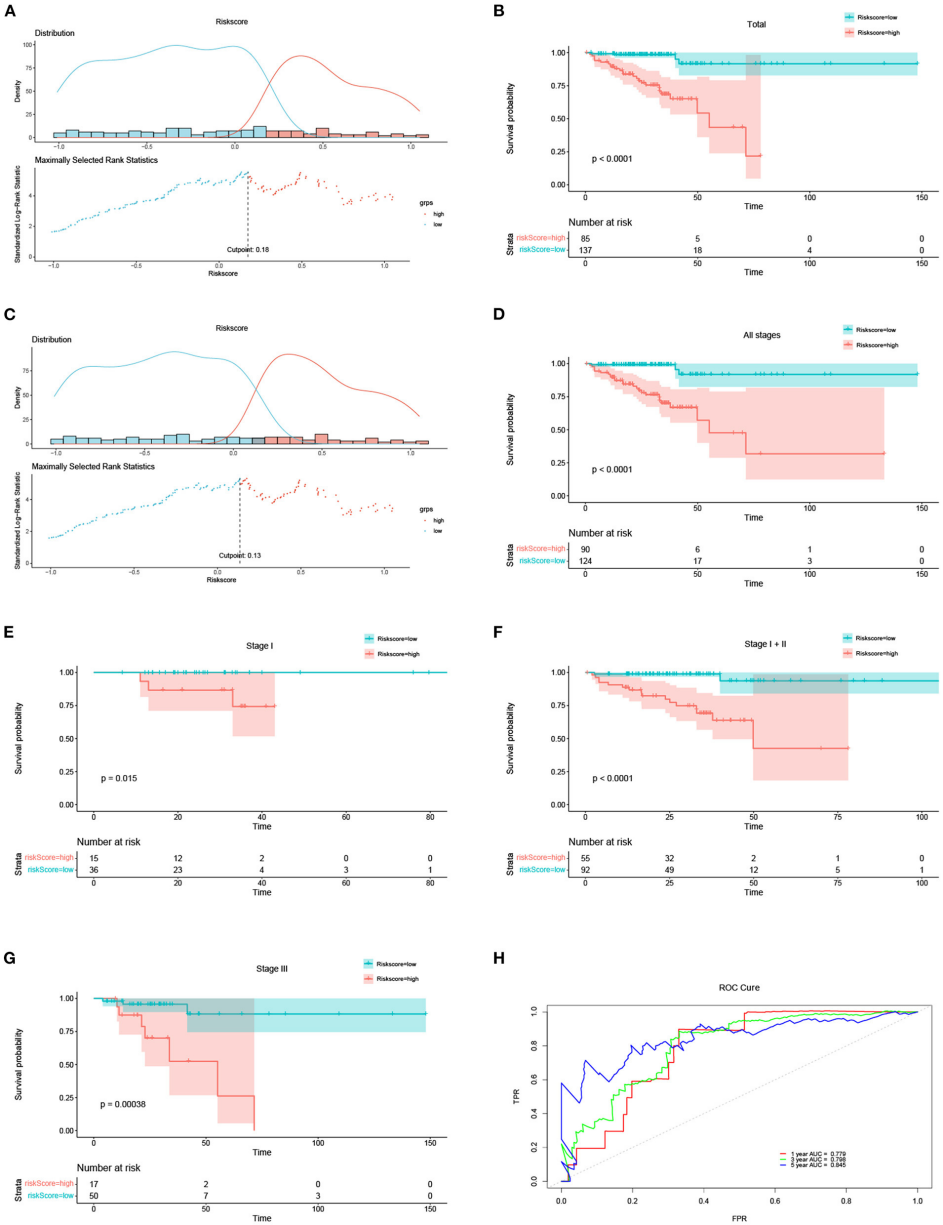
The risk score results acted as an independent prognostic factor in DFS. **(A)** Optimal cut-off of the total 222 DFS patients and distribution of high- and low-risk groups based on the optimal cut-off of 0.18. **(B)** Survival curves of the total 222 DFS patients under the optimal cut-off of 0.18. **(C)** Optimal cut-off of the total 214 DFS patients at different stages (including stage I, II and III) and distribution of high- and low-risk groups based on the optimal cut-off of 0.13. **(D)** Survival curves of all 214 DFS patients under the optimal cut-off of 0.13. **(E–G)** Survival curves of the DFS patients at stages I, I + II, and III, respectively. **(H)** ROC of 1-, 3-, and 5-years predicted by the risk score.

Kaplan-Meier (KM) survival analysis was conducted for the risk score. The optimal risk score cut-off point in 222 DFS patients with DFS status and time records was 0.18 as illustrated in Figure 2A. Based on this best cut-off point, we divided the 222 DFS patients into high- and low-risk groups, the distribution of which was illustrated in Figure 2A. The survival curves of the 222 DFS patients under the optimal cut-off point were depicted in Figure 2B (*P* < 0.0001). The optimal cut-off point for the risk score in all 214 patients with stages I, II, and III was 0.13, as illustrated in Figure 2C. Based on this optimal cut-off point, we separated all 214 DFS patients into high- and low-risk groups, the distribution of which was depicted in Figure 2C. The survival curves of the 214 patients are shown in Figure 2D (*P* < 0.0001). The survival curves of the patients with stage I, I + II, and III are depicted in Figures 2E–G with *P* = 0.015, *P* < 0.0001, and *P* = 0.00038, respectively. All survival curves indicated that the risk score was an independent prognostic parameter, and the low-risk group had a favorable prognosis. As an independent prognostic parameter, the predictive performance of the risk score was represented by the ROC in Figure 2H. The AUCs of 1-, 3-, and 5-year predicted by the risk score were 0.779, 0.798, and 0.845, respectively. The OS results for TCGA are included in Supplementary Figure 4. The survival curves of DFS and OS for the validation data set are also included in the Supplementary Figures 5, 6.

### The Prognostic Model as an Independent Clinical Parameter

**Figure 3:**
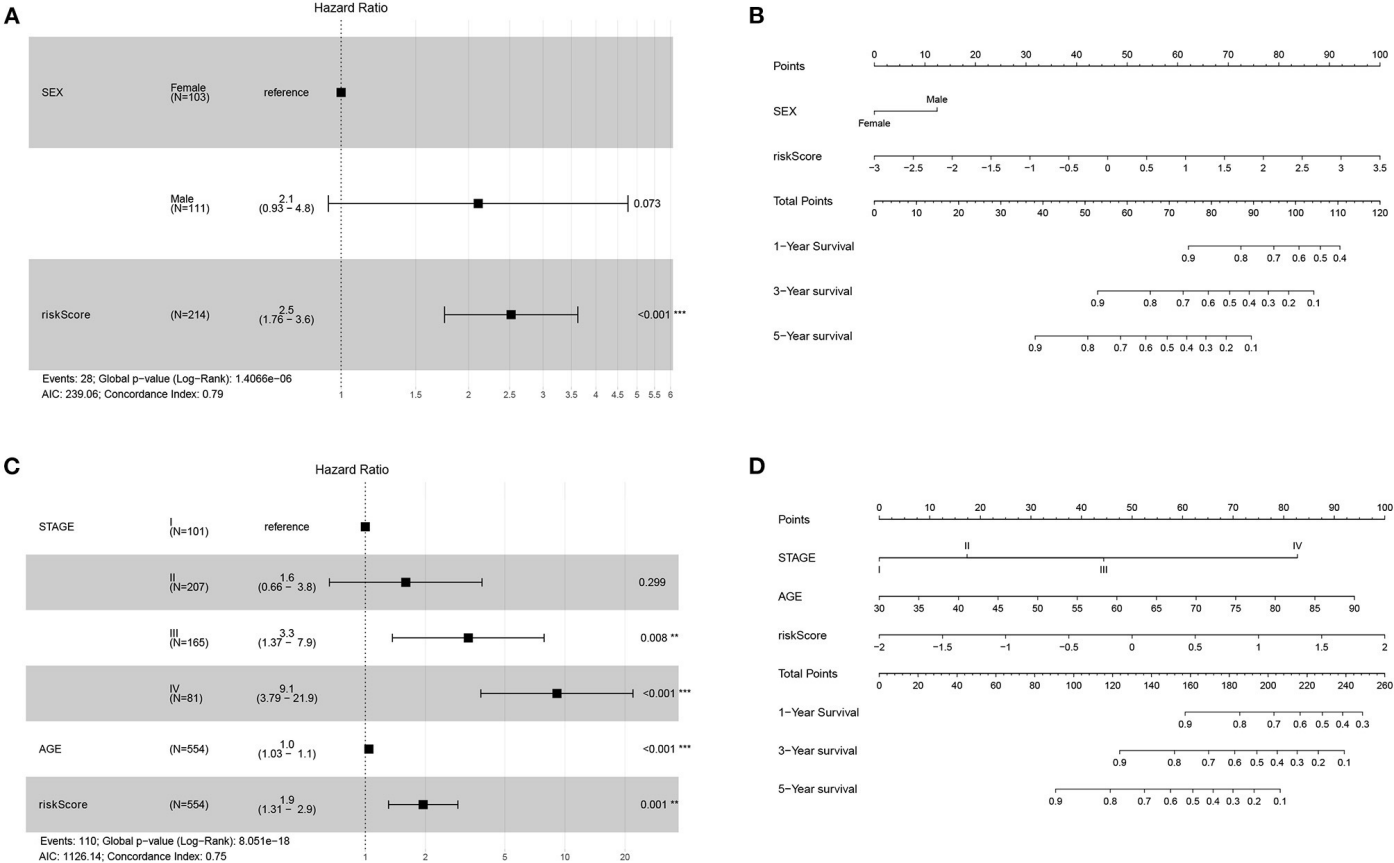
Forest plots and nomograms for DFS and OS in TCGA-COAD. **(A)** Forest plot for DFS in TCGA-COAD. **(B)** Nomogram for DFS in TCGA-COAD. **(C)** Forest plot for OS in TCGA-COAD. **(D)** Nomogram for OS in TCGA-COAD. **P < 0.01; ***P < 0.001.

Univariate Cox regression analysis of the 214 DFS patients included in the TCGA-COAD data set indicated that sex and our prognostic model were significantly correlated. Multivariate Cox regression analysis revealed that this prognostic model was an independent prognostic parameter of DFS (Figure 3A). Among the 554 OS patients included in the TCGA-COAD data set, the prognostic model was also shown to be an independent prognostic parameter of OS (Figure 3C), which was consistent with the results from the TCGA-COAD DFS cohort. To make the proposed approach more practical for clinicians, nomograms for both DFS and OS were developed to predict survival probability (Figures 3B,D). The C-indices of DFS and OS were 0.786 and 0.748, separately. The results for the validation data set are included in the Supplementary Figure 7.

### Validation of the Nomogram

**Figure 4:**
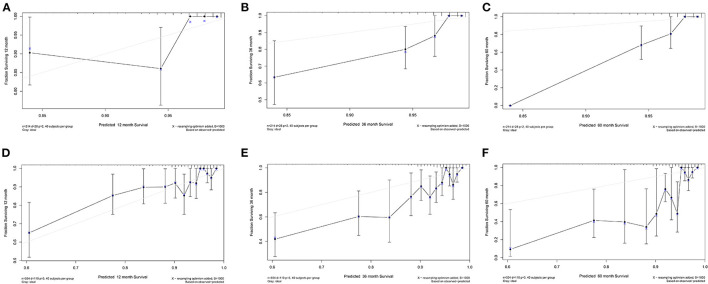
Calibration curve for TCGA-COAD. **(A–C)** Calibration curve of 1-, 3-, and 5-year survival for DFS patients in TCGA. **(D–F)** Calibration curve of 1-, 3-, and 5-year survival for OS patients in TCGA.

The calibration curve for TCGA-COAD was plotted to validate the predictive performance of the nomogram, which was presented in Figure 4. The calibration curves illustrated in Figures 4A–C represent the 1-, 3-, and 5-year survival of DFS patients in TCGA. The x-axis suggests the nomogram prediction probabilities and the y-axis indicates the observed rates. The dashed line denotes the reference line. The closer the calibration curve was to the reference line, the better the predictive performance of the nomogram. The same results for OS patients in TCGA were illustrated in Figures 4D,E. The calibration curves for the validation dataset of GSE39582 were shown in Supplementary Figure 8.

**Figure 5:**
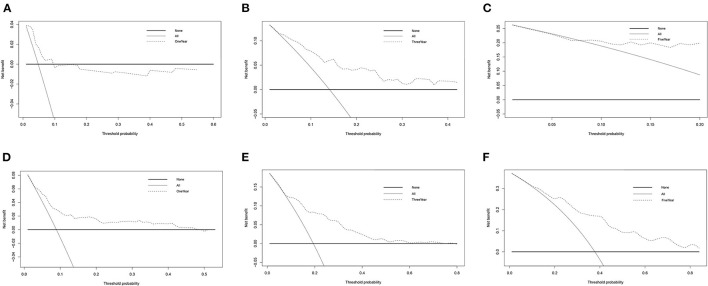
DCA for TCGA-COAD. **(A–C)** DCA of 1-, 3-, and 5-year survival for DFS patients in TCGA. **(D–F)** DCA of 1-, 3- and 5-year survival for OS patients in TCGA.

In DCA, the y-axis denotes the net benefits and the x-axis indicates the threshold probability. The gray diagonal line in Figure 5 denotes the hypothesis that all patients have 1-, 3-, and 5-year survival rates. The black horizontal solid lines represent the surmise that no patients have 1-, 3-, or 5-year survival rate. The 1-, 3-, and 5-year DCA for DFS patients in TCGA were illustrated in Figures 5A–C. The higher the net benefit, the better the nomogram. The same results for OS patients in TCGA were shown in Figures 5D–F. Additionally, the DCA for the validation dataset of GSE39582 is shown in Supplementary Figure 9.

### External Validation Using the Online Database

**Figure 6:**
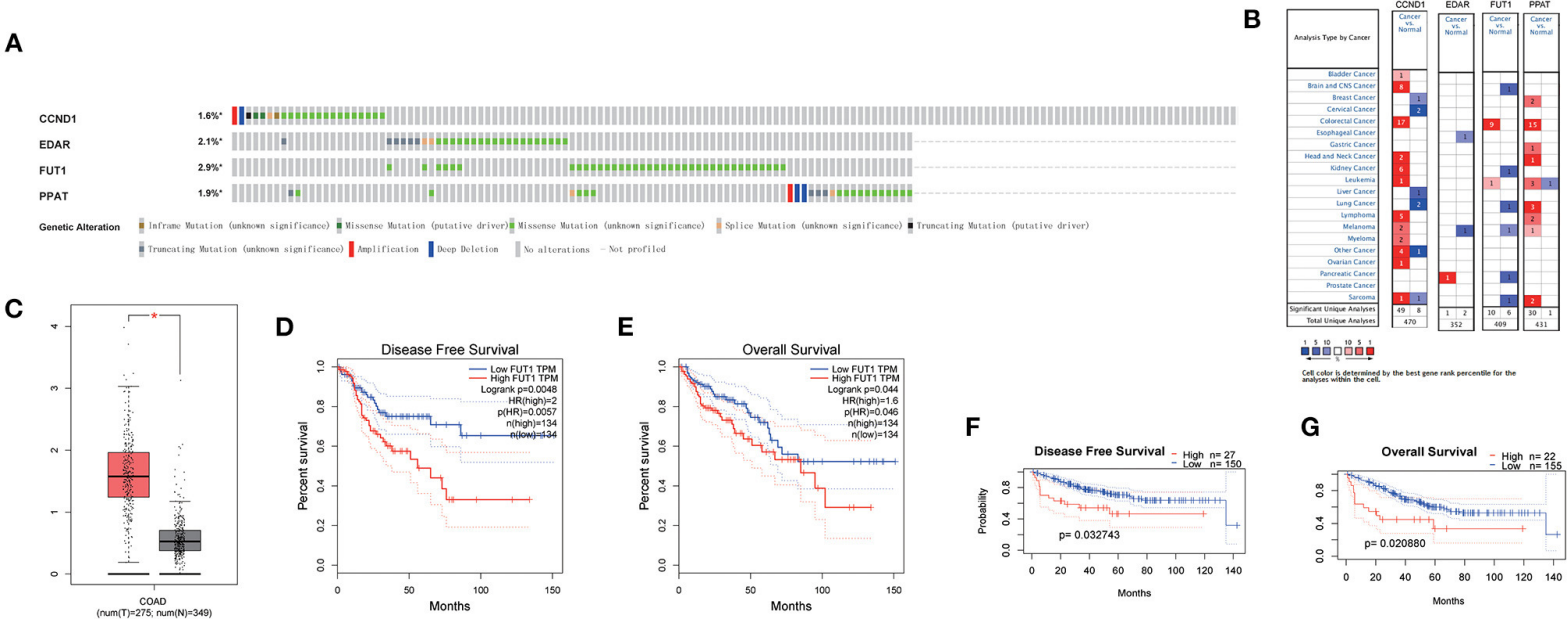
Expression and genetic alterations of the four predictive genes. **(A)** Genetic alterations of the four genes. The data were obtained from the cBioportal in COAD. **(B)** Expression profiles of the four genes in the Oncomine database. **(C)** FUT1 expression in COAD and normal tissue in GEPIA. **(D)** Prognostic value of FUT1 expression in DFS in GEPIA. **(E)** Prognostic value of FUT1 expression in OS in GEPIA. **(F)** Prognostic value of FUT1 expression in DFS in GSE17536 in PrognoScan. **(G)** Prognostic value of FUT1 expression in OS in GSE17536 in PrognoScan. *P < 0.05.

In the cBioportal for Cancer Genomics website, FUT1 exhibited the most frequent genetic alterations (2.9%) among the four genes of the prognostic model, with missense mutation being the most common alteration (Figure 6A). Consistent with our results, CCND1, FUT1, and PPAT were found to be significantly overexpressed in tumors, whereas EDAR was not significantly expressed in COAD in Oncomine (Figure 6B). FUT1 was also found to be significantly overexpressed in COAD tumors compared to normal tissues in the GEPIA database (23) (Figure 6C). Survival analyses were carried out by the GEPIA database. Patients with high FUT1 expression displayed remarkably shorter DFS and OS in GEPIA (Figures 6D,E); however, CCND1, EDAR, and PPAT showed no significant differences in DFS and OS (Supplementary Figure 10). Further, FUT1 was found to be consistent with the DFS and OS results of GSE17536 in PrognoScan (Figures 6F,G). Taken together, our findings indicated that FUT1 was an aberrantly expressed gene and high FUT1 expression could be used as a predictor of adverse outcomes.

### si-FUT1 Inhibited Cell Migration and Induced Apoptosis in Colon Cancer Cells

**Figure 7:**
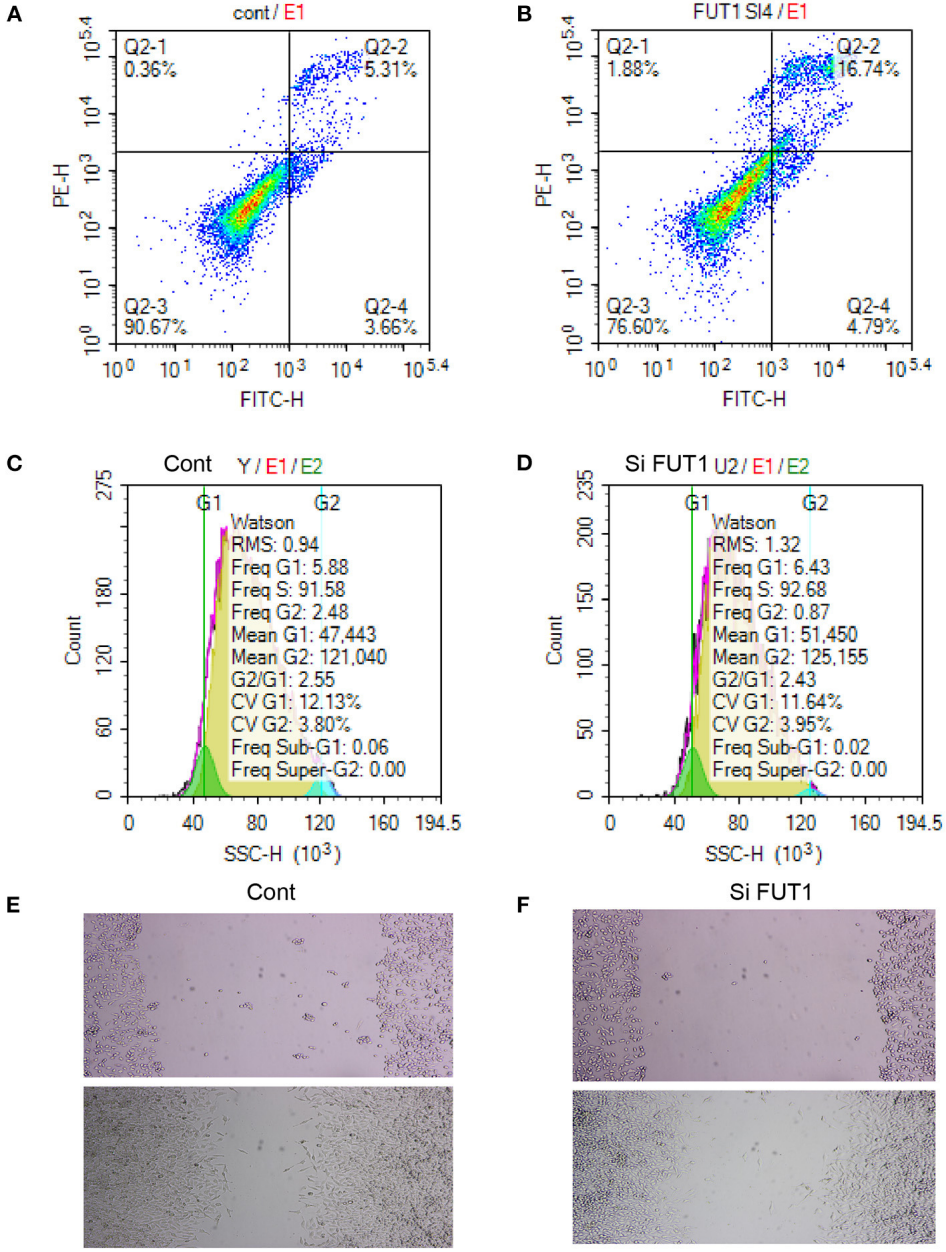
Apoptosis and cycle assay and cell transfection experiment. **(A)** Flow cytometry assay in NC cells. **(B)** Flow cytometry assay in HCT-116 cells. **(C)** Cell cycle in NC cells. **(D)** Cell cycle in HCT-116 cells. **(E)** Cell migration in NC cells. **(F)** Cell migration in HCT-116 cells.

A flow cytometry assay was performed to examine the effect of FUT1 on cell apoptosis and the cell cycle. Transfection with si-FUT1 significantly raised the apoptotic rate of HCT-116 cells compared with that of NC cells (Figures 7A,B), and significantly reduced the number of cells in the G2 phase (Figures 7C,D). Next, cell migration was detected using the streak method. Transfection with si-FUT1 significantly inhibited the cell migration rate of HCT-116 cells compared to that of NC cells (Figures 7E,F).

## Discussion

Metabolic reprogramming is considered as a new critical feature of malignancy (1). Some potential molecules regulating abnormal metabolism have been tested in preclinical or clinical investigations (24). The interactions between bile acids, cholesterol metabolites, and colonic epithelial cells may be relevant in colon carcinogenesis (25). Meanwhile, big data analysis is an important tool for Prediction task (26–30), and disease forecasting is a vital part of the medical research (31, 32). However, the role and mechanisms of metabolic-related genes in COAD remain unclear. The present study was thus conducted to further explore the association of metabolic genes with prognosis in patients with COAD.

Big data and machine learning algorithms have been widely used in basic and clinical medicine research, such as antlion re-sampling based deep neural network model for classification of imbalanced multimodal stroke dataset, and deep neural networks to predict diabetic retinopathy (32, 33). High-throughput multimodal massive medical data is just right for research with big data and machine learning algorithms. The blockchain-enabled internet of medical things (IoMT) can provide strong trust establishment and ensure the traceability of data sharing in the IoMT networks (34). Due to the emergence of heterogeneous IoMT, large volumes of patient data are dispatched to central cloud servers for disease analysis and diagnosis (29). Many publicly available data resources and analytical tools are available for research. Analysis methods including survival analysis, nomogram and data visualization have also been developed with corresponding R packages for researchers to use. Our work used LASSO machine learning algorithms as survival analysis and prediction tool. Multiple data sources and analytical tools in a way that leverages data from previous studies and yields valuable results even when clinical trials are not yet readily available. As more data resources and analysis tools are developed, we believe that big data and machine learning algorithms will be more widely used in medical research.

In our research, we comprehensively probed the expression of 62 metabolic genes in COAD tumor tissues and the relationships between recurrent patients and DFS and OS. A new prognostic model including four metabolic genes associated with DFS was first established based on the TCGA dataset and validated using the GSE39582 dataset. The validity of the novel signature was shown in the training, validating, and stage subgroups. The signature exhibited a robust prognostic capacity, especially for the short-term survival of patients with COAD. Besides, the OS of patients with high-risk was shorter than that of patients with low-risk in the TNM stage. Multivariate Cox regression analysis indicated that our prognostic model was an independent prognostic parameter for DFS and OS (HR > 1, *P* < 0.001). ROC curve analysis verified the predictive power of the features. Additionally, the forest plot suggested that the risk score was an independent parameter in the multivariate Cox regression analyses. Moreover, a nomogram was built to predict 1-, 3-, and 5-year DFS rates. The efficacy of the nomogram was verified in validation cohorts, and the calibration plots and DCA showed that the precision of the nomogram was good. Therefore, our nomogram may provide simple and accurate prognostic predictions for COAD.

In the cBioportal for Cancer Genomics website, FUT1 possessed the most frequent genetic alterations (2.9%) among the four genes of the prognostic model. FUT1 was also found to be obviously overexpressed at the mRNA level in COAD tumors compared to normal tissues in the GEPIA database (Figure 6C). Patients with high FUT1 expression displayed remarkably shorter DFS and OS in the GEPIA to low FUT1 expression (Figures 6D,E). Moreover, the FUT1 results were consistent with the DFS and OS results in GSE17536 in PrognoScan (Figures 6F,G). Taken together, our findings indicated that FUT1 was an aberrantly expressed gene and therefore high FUT1 expression could be used as a predictor of adverse outcomes.

FUT1 encodes a Golgi stack membrane protein that participates in the synthesis of a precursor of the H antigen. Gene expression profiling analysis unexpectedly showed a significant number of up- and down-regulated metabolism-associated FUT1 genes in nasopharyngeal carcinoma (35). Additionally, FUT1 plays an important role in a regulatory mechanism involving fucosylation through which glucose restriction promotes cancer stemness to drive tumor recurrence and drug resistance. FUT1 overexpression is a poor prognostic indicator of hepatocellular carcinoma (36). We also examined the function of FUT1 in cell apoptosis and cell cycle and performed a flow cytometry assay. Transfection with si-FUT1 significantly raised the apoptotic rate of HCT-116 cells and obviously reduced the number of cells in the G2 phase while also significantly inhibiting the cell migration rate of HCT-116 cells compared to that of NC cells. Therefore, si-FUT1 inhibited cell migration and induced apoptosis in colon cancer cells. FUT1 overexpression may thus be a new prognostic marker and therapeutic target of COAD tumors.

Functional analyses also revealed enriched metabolism-related pathways. The top categories were critically important in COAD patients and strongly associated with metabolism, particularly the G1/S transition of the mitotic cell cycle. Correlation analysis between the genes in the risk score DFS model expression and six types of infiltrating immunocytes suggested that CD8+ T-cells and B-cells may be significantly related with COAD in both DFS and OS.

Immunocytes are essential ingredients of the tumor microenvironment. These immunocytes differentiate into subsets with distinct effects, and metabolic reprogramming participates in this process (37). These immunocytes in the tumor microenvironment have metabolic characteristics that differ from those in non-tumor tissues (38). OS was dramatically worse in patients with low levels of CD8+ T-cell infiltrating than those with high levels of CD8+ T-cell infiltrating. The survival rate in patients with high CD8+ T cell infiltrating was 100%. Peritumoral CD8+ T-cell infiltration has an anti-tumor effect in patients with colorectal cancer (39). CD8+ T cell expansion and function rely on glycolysis; however, the mechanisms underlying CD8+ T cell metabolism remain unclear (40). We previously demonstrated that increasing B-cell a infiltrating, clonal expansion, and mutational frequency from the cecum to the sigmoid colon were linked to an increasing number of reactive bacterial species (41). Numerous B-cell clones distribute into two broad networks: one includes the blood, bone marrow, spleen, and lung, whereas the other is distributed to digestive tract, including colorectal tissues (42). B-cell clonal lineages is a basis for investigations on tissue-based immunity, including infection, vaccine response, autoimmunity, and tumor (42). Metabolic reprogramming of tumor cells and the tumor microenvironment are up-and-coming as essential characteristics affecting tumor development, metastasis, and response to treatments (43). A better understanding of metabolic communications among tumor cells, intestinal flora, and immunocyte populations will open new ways for identifying strategies to boost anti-tumoral immune responses in COAD patients.

Although our work has yielded many meaningful results through bioinformatics methods, these results still need to be further validated by preclinical studies and clinical trials. The use of numerous data sources and analytical tools also makes it more difficult to understand the research process and results. The interpretation of the results of big data studies is also an area prone to controversy. Therefore, there is still work to be done in predicting the prognosis of patients with COAD. For example, more patients and clinical characteristics of patients should be included in further studies.

## Conclusions

In summary, we identified a novel signature comprised of four metabolic genes that could precisely predict the prognosis of patients with COAD. Metabolic-related signatures may have a potential role in the anti-tumor process and serve as therapeutic targets for COAD.

## Data Availability Statement

The original contributions presented in the study are included in the article/Supplementary Material, further inquiries can be directed to the corresponding author/s.

## Author Contributions

CY and XW designed the article. YZ, RW, and CY collected and evaluated the data and wrote the first draft of the manuscript. All authors reviewed the manuscript, contributed to the interpretation of the results, wrote the final draft of the manuscript, and read and approved the final version of the manuscript.

## Funding

This work was supported by National Key R&D Program of China (2017YFC1308800).

## Conflict of Interest

The authors declare that the research was conducted in the absence of any commercial or financial relationships that could be construed as a potential conflict of interest.

## Publisher's Note

All claims expressed in this article are solely those of the authors and do not necessarily represent those of their affiliated organizations, or those of the publisher, the editors and the reviewers. Any product that may be evaluated in this article, or claim that may be made by its manufacturer, is not guaranteed or endorsed by the publisher.

## Supplementary Material

Supplementary Figure 1 Study flowchart.

Supplementary Figure 2 Correlation between the expression of the genes in the risk score model of DFS and the levels of immune cell infiltration in COAD samples, as investigated by TIMER analysis. **(A)** CCND1. **(B)** EDAR. **(C)** FUT1. **(D)** PPAT.

Supplementary Figure 3 Correlation between the expression of the genes in the risk score model of OS and immune infiltrating levels in COAD samples, as assessed by TIMER analysis. **(A)** CCNB1. **(B)** CDC6. **(C)** FUT1. **(D)** GPD1L. **(E)** MAD2L1. **(F)** MMP1. **(G)** SPP1. **(H)** TKT.

Supplementary Figure 4 The risk score results acted as an independent prognostic factor in OS of TCGA. **(A)** Optimal cut-off of the total 566 overall survival patients and distribution of high-risk and low-risk groups based on the optimal cut-off of 0.38. **(B)** Survival curves of the 554 overall survival patients under the optimal cut-off of 0.38. **(C–E)** Survival curves of the patients at stage I + II, stage III, and stage IV, respectively.

Supplementary Figure 5 Survival curves of DFS in the validation data set of GSE39582. **(A)** Survival curves of 532 DFS patients at different stages (stage I, II, III, and IV). **(B)** Survival curves of DFS patients at stage I. **(C)** Survival curves of DFS patients at stage I + II. **(D)** Survival curves of DFS patients at stage III.

Supplementary Figure 6 OS curves from the GSE39582 validation data set. **(A)** Survival curves of 569 overall surviving patients at different stages (stage I, II, III, and IV). **(B)** Survival curves of disease-free surviving patients at stage III. **(C)** Survival curves of disease-free surviving patients at stage IV.

Supplementary Figure 7 Forest plots and nomograms for DFS and OS in the GSE39582 validation data set. **(A)** Forest plot for DFS in GSE39582. **(B)** Nomogram for DFS in GSE39582. **(C)** Forest plot for overall survival in GSE39582. **(D)** Nomogram for overall survival in GSE39582.

Supplementary Figure 8 Calibration curve for the GSE39582 validation data set. **(A–C)** Calibration curve of 1-, 3-, and 5-year survival for disease-free surviving patients in GSE39582. **(D–F)** Calibration curve of 1-, 3-, and 5-year survival for overall surviving patients in GSE39582.

Supplementary Figure 9 DCA for validation of data set GSE39582. **(A–C)** DCA of 1-, 3- and 5-year survival for disease-free surviving patients in GSE39582. **(D–F)** DCA of 1-, 3-, and 5-year survival for overall surviving patients in GSE39582.

Supplementary Figure 10 Prognostic value of gene expression in GEPIA. **(A)** CCND1 in DFS. **(B)** CCND1 in OS. **(C)** EDAR in DFS. **(D)** PPAT in OS. **(E)** PPAT in DFS. **(F)** PPAT in OS.

Supplementary Table 1 List of metabolic-related genes.
