# Supplementary figures and images for: Identification of a Four-Gene Metabolic Signature to Evaluate the Prognosis of Colon Adenocarcinoma Patients
Source: Front Public Health. 2022 Apr 7;10:860381. doi: 10.3389/fpubh.2022.860381 (PMC9021388; doi:10.3389/fpubh.2022.860381)

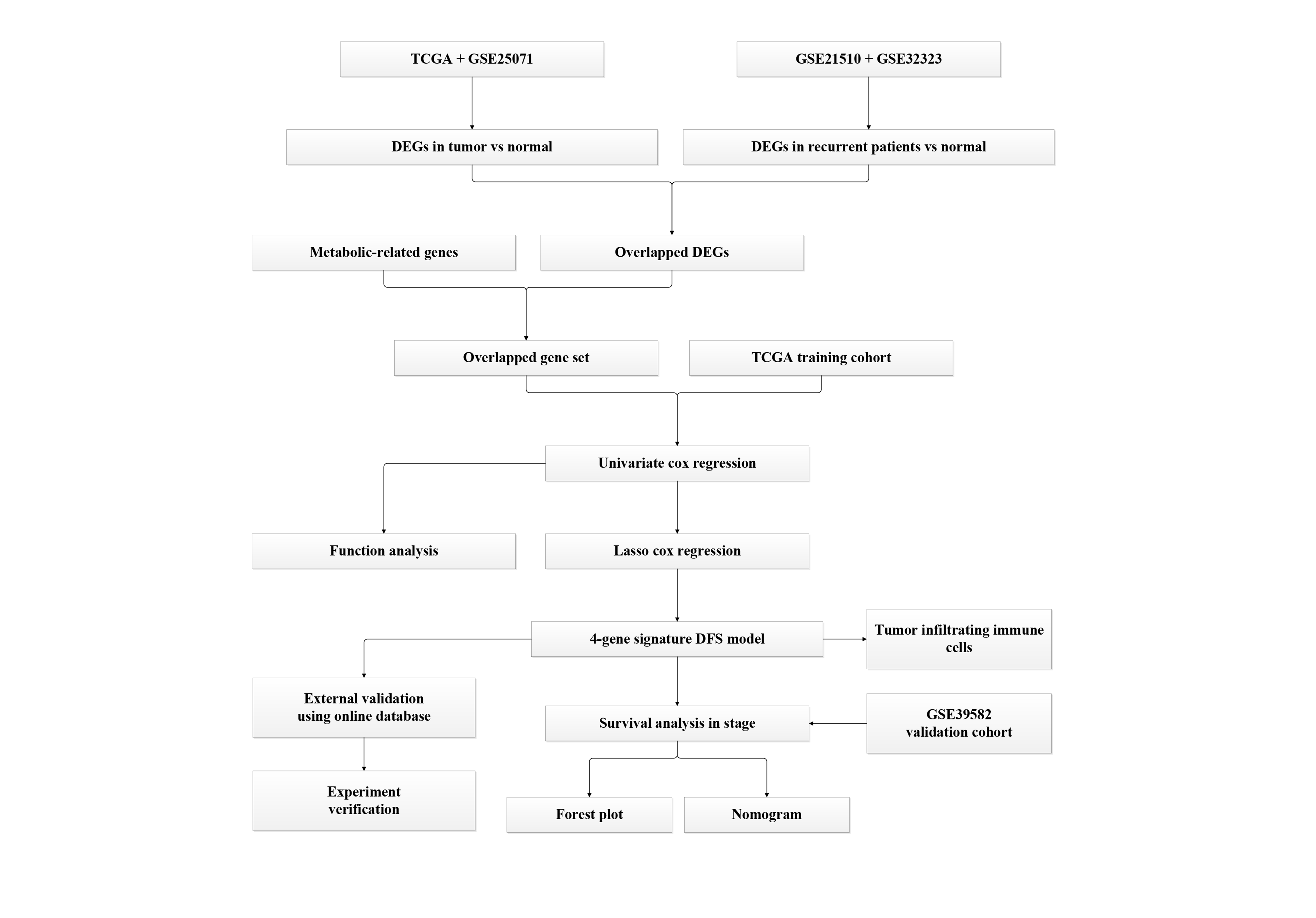

Supplement: Supplementary Figure 1 — Study flowchart. [file Image_1.TIF]

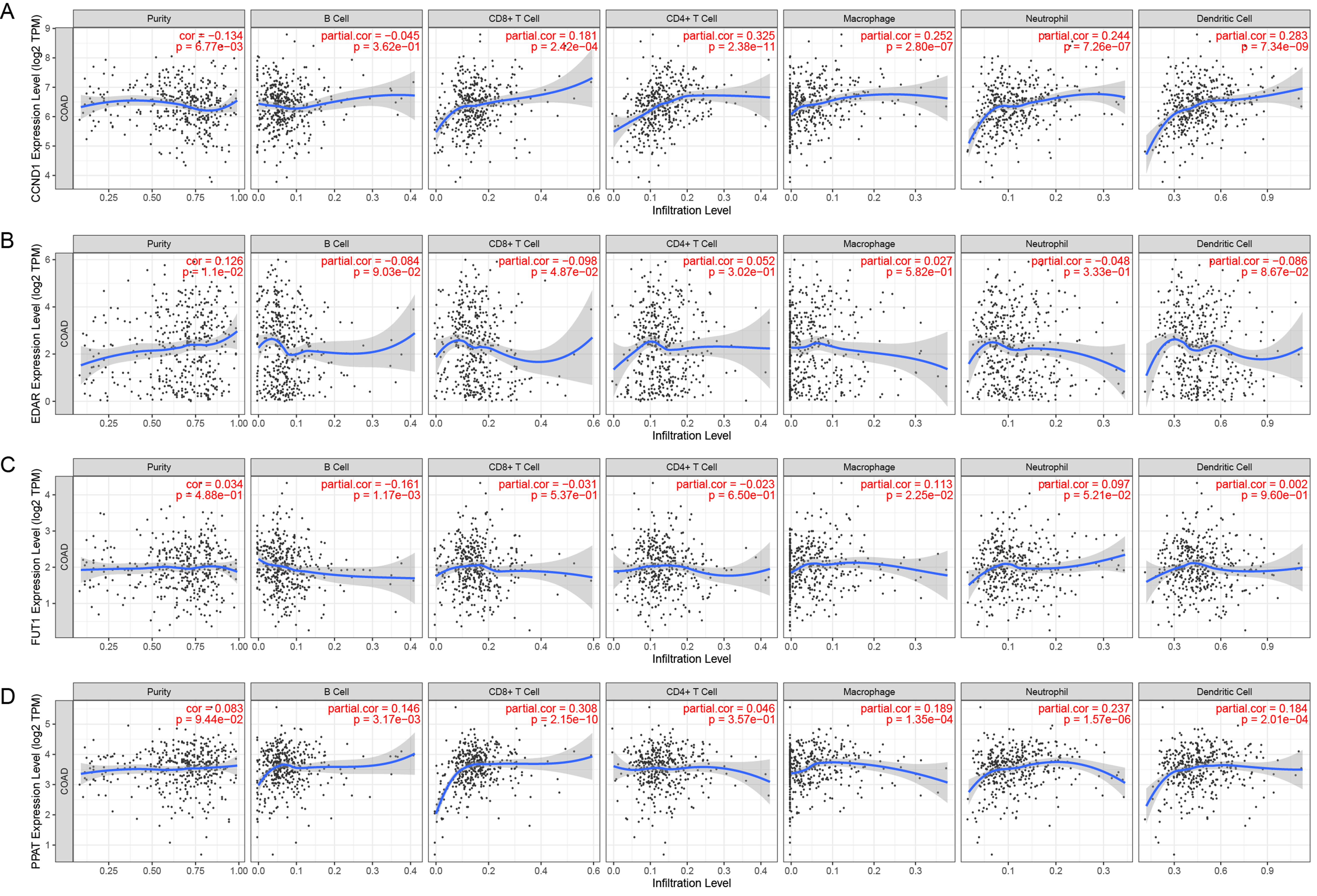

Supplement: Supplementary Figure 2 — Correlation between the expression of the genes in the risk score model of DFS and the levels of immune cell infiltration in COAD samples, as investigated by TIMER analysis. (A) CCND1. (B) EDAR. (C) FUT1. (D) PPAT. [file Image_2.TIF]

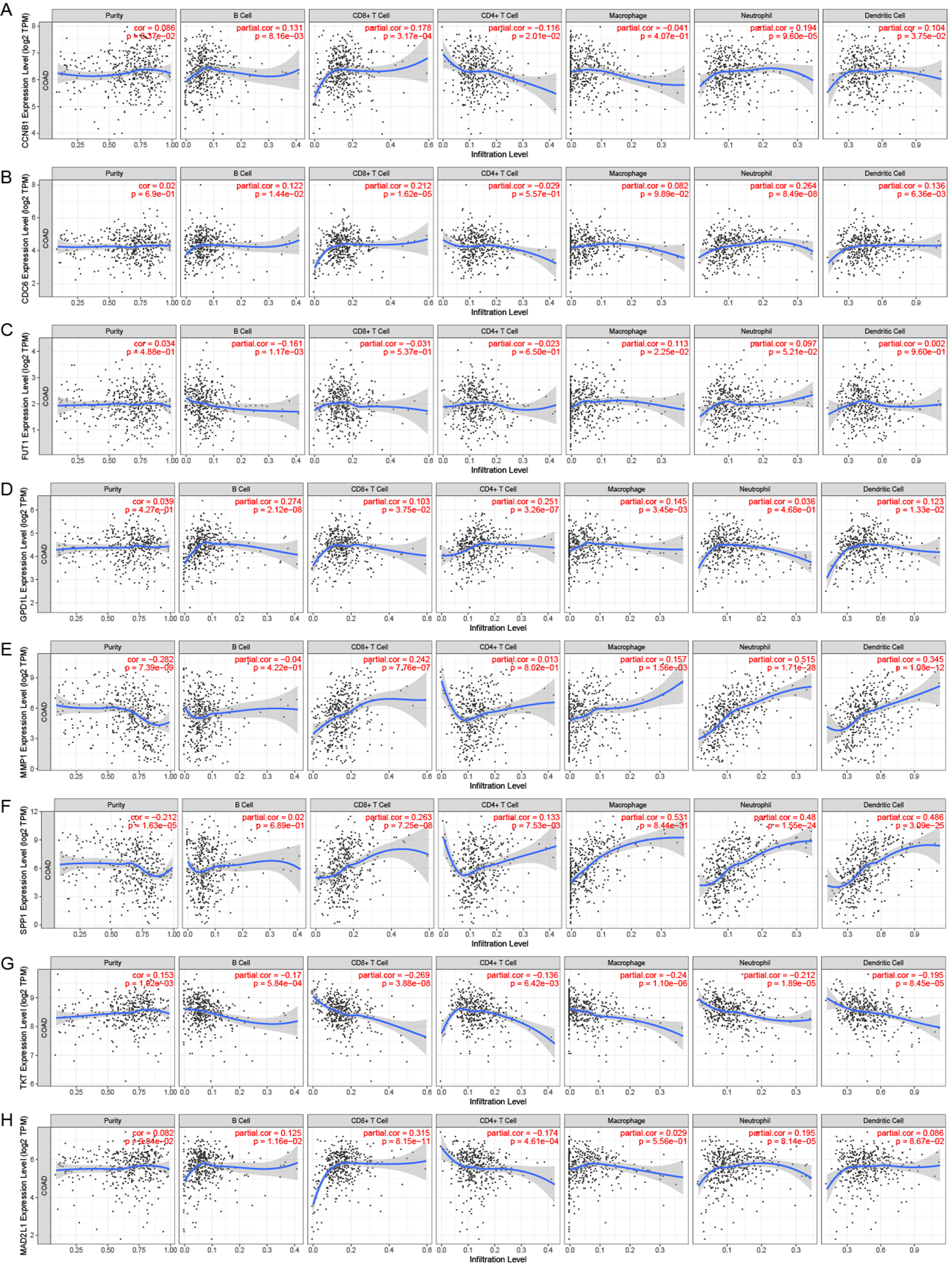

Supplement: Supplementary Figure 3 — Correlation between the expression of the genes in the risk score model of OS and immune infiltrating levels in COAD samples, as assessed by TIMER analysis. (A) CCNB1. (B) CDC6. (C) FUT1. (D) GPD1L. (E) MAD2L1. (F) MMP1. (G) SPP1. (H) TKT. [file Image_3.TIF]

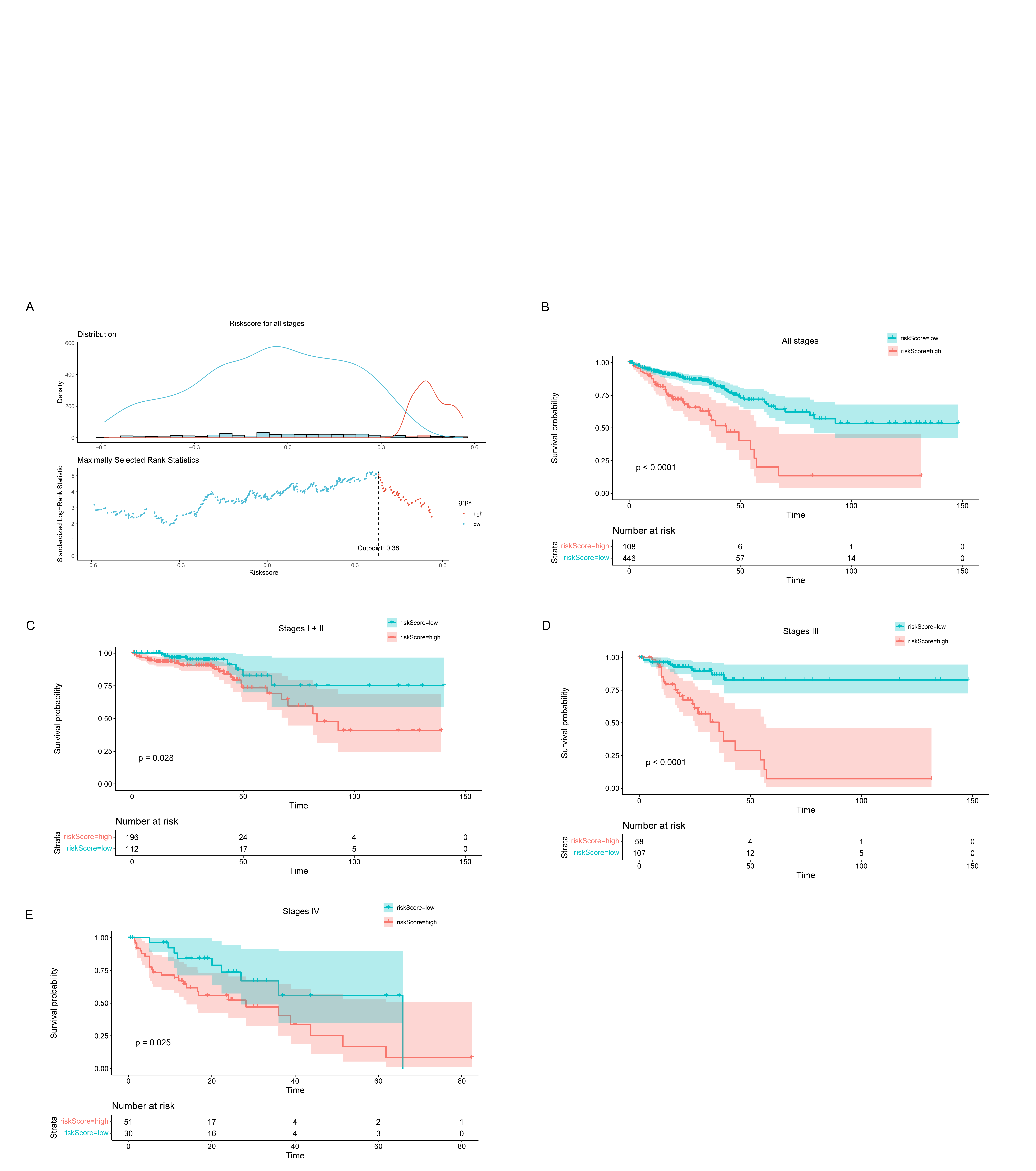

Supplement: Supplementary Figure 4 — The risk score results acted as an independent prognostic factor in OS of TCGA. (A) Optimal cut-off of the total 566 overall survival patients and distribution of high-risk and low-risk groups based on the optimal cut-off of 0.38. (B) Survival curves of the 554 overall survival patients under the optimal cut-off of 0.38. (C–E) Survival curves of the patients at stage I + II, stage III, and stage IV, respectively. [file Image_4.TIF]

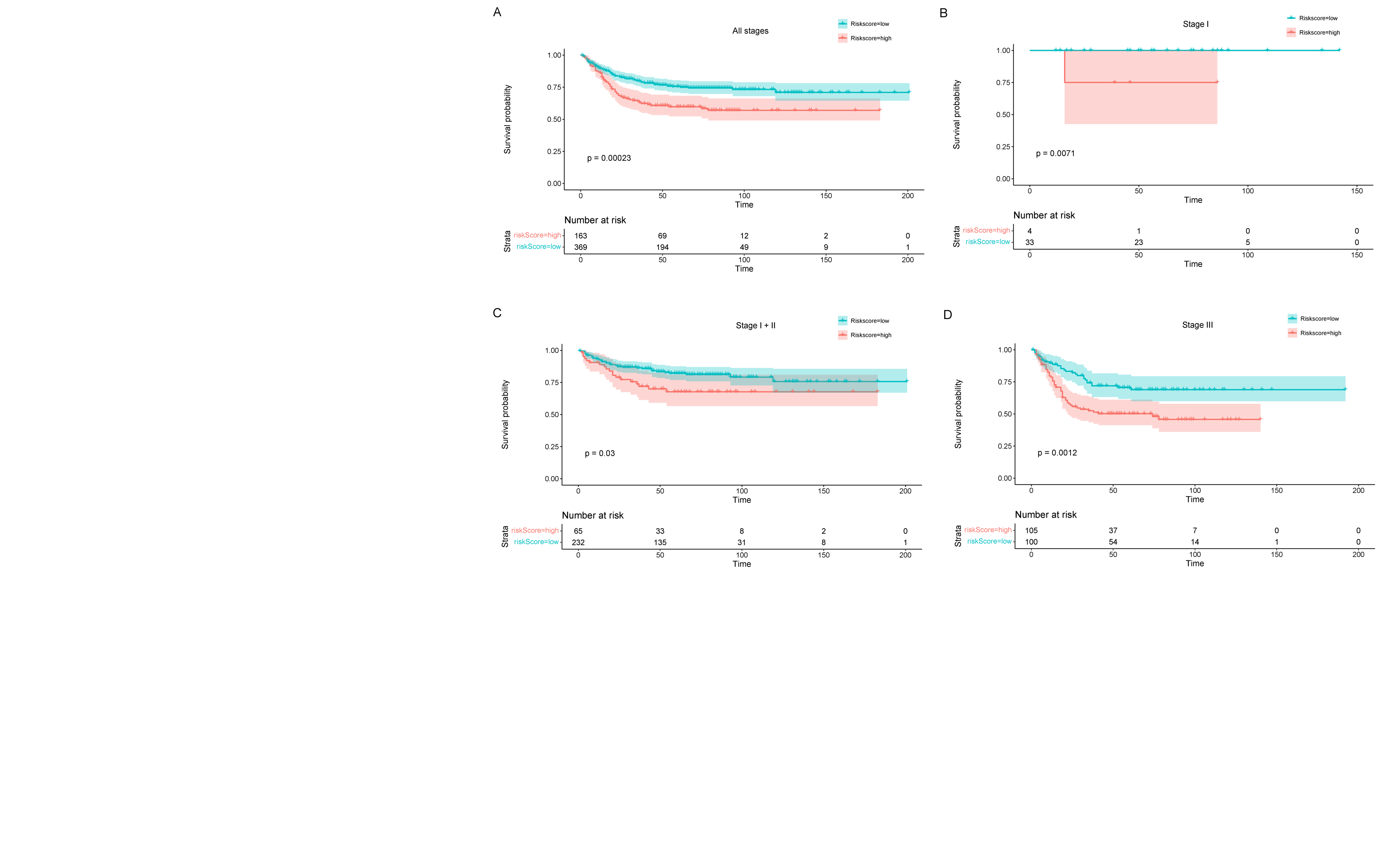

Supplement: Supplementary Figure 5 — Survival curves of DFS in the validation data set of GSE39582. (A) Survival curves of 532 DFS patients at different stages (stage I, II, III, and IV). (B) Survival curves of DFS patients at stage I. (C) Survival curves of DFS patients at stage I + II. (D) Survival curves of DFS patients at stage III. [file Image_5.TIF]

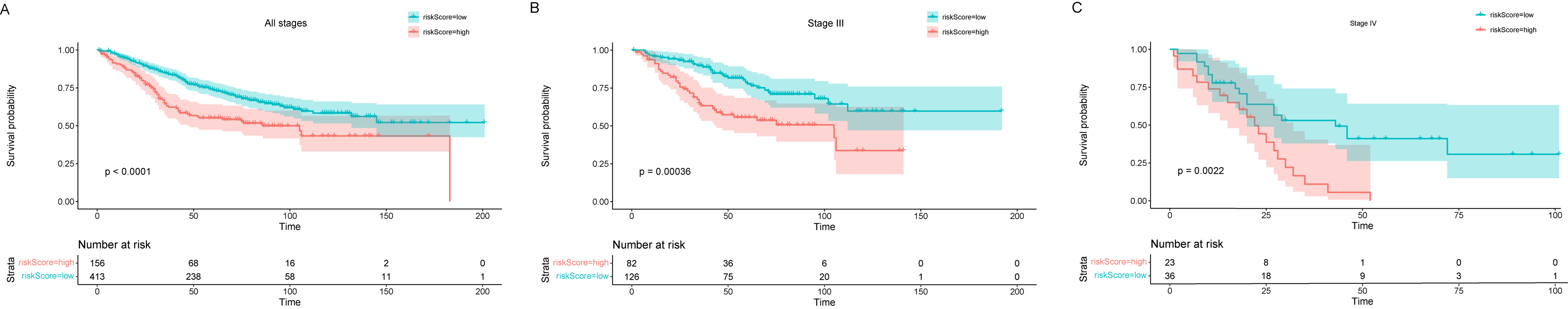

Supplement: Supplementary Figure 6 — OS curves from the GSE39582 validation data set. (A) Survival curves of 569 overall surviving patients at different stages (stage I, II, III, and IV). (B) Survival curves of disease-free surviving patients at stage III. (C) Survival curves of disease-free surviving patients at stage IV. [file Image_6.TIF]

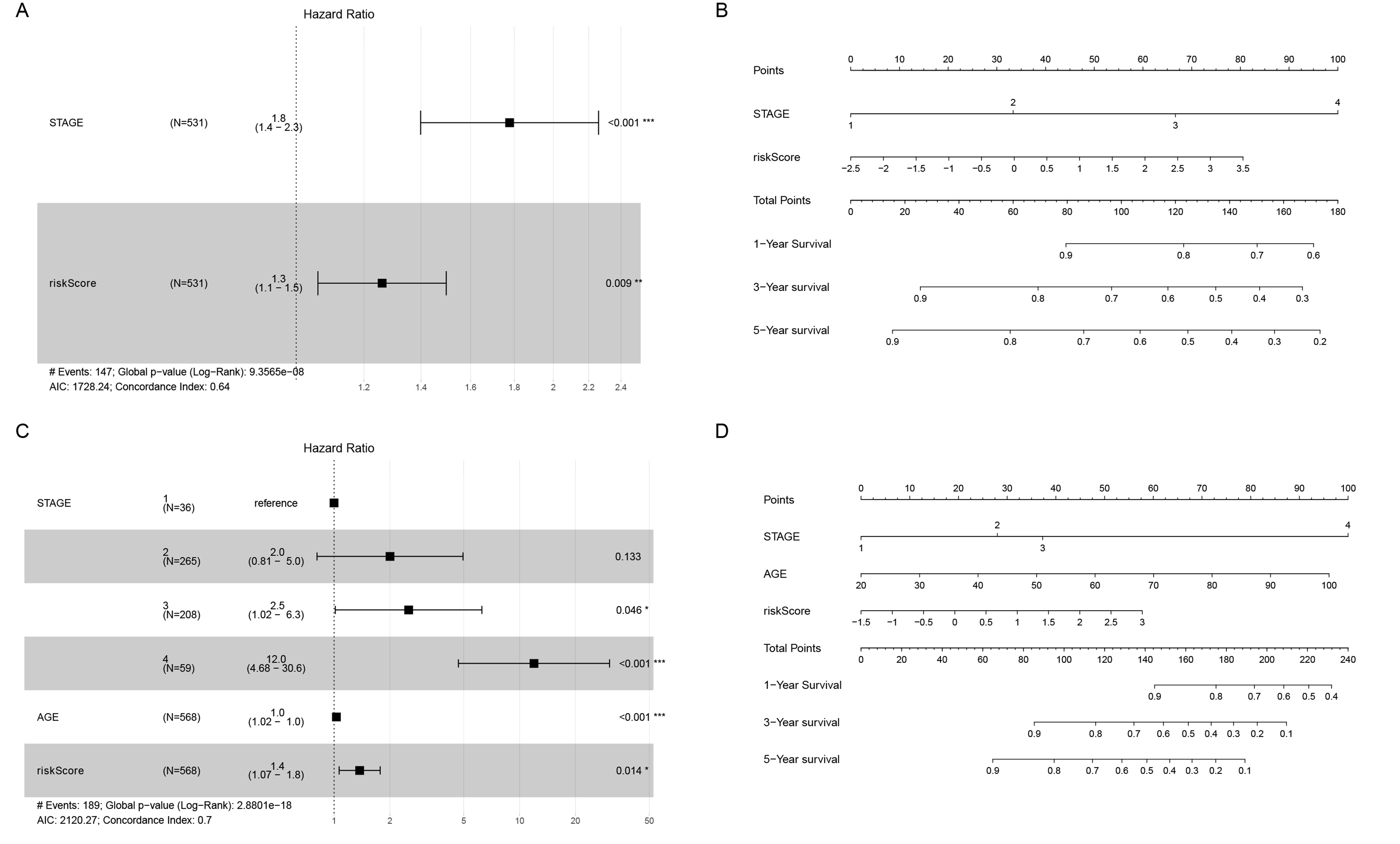

Supplement: Supplementary Figure 7 — Forest plots and nomograms for DFS and OS in the GSE39582 validation data set. (A) Forest plot for DFS in GSE39582. (B) Nomogram for DFS in GSE39582. (C) Forest plot for overall survival in GSE39582. (D) Nomogram for overall survival in GSE39582. [file Image_7.TIF]

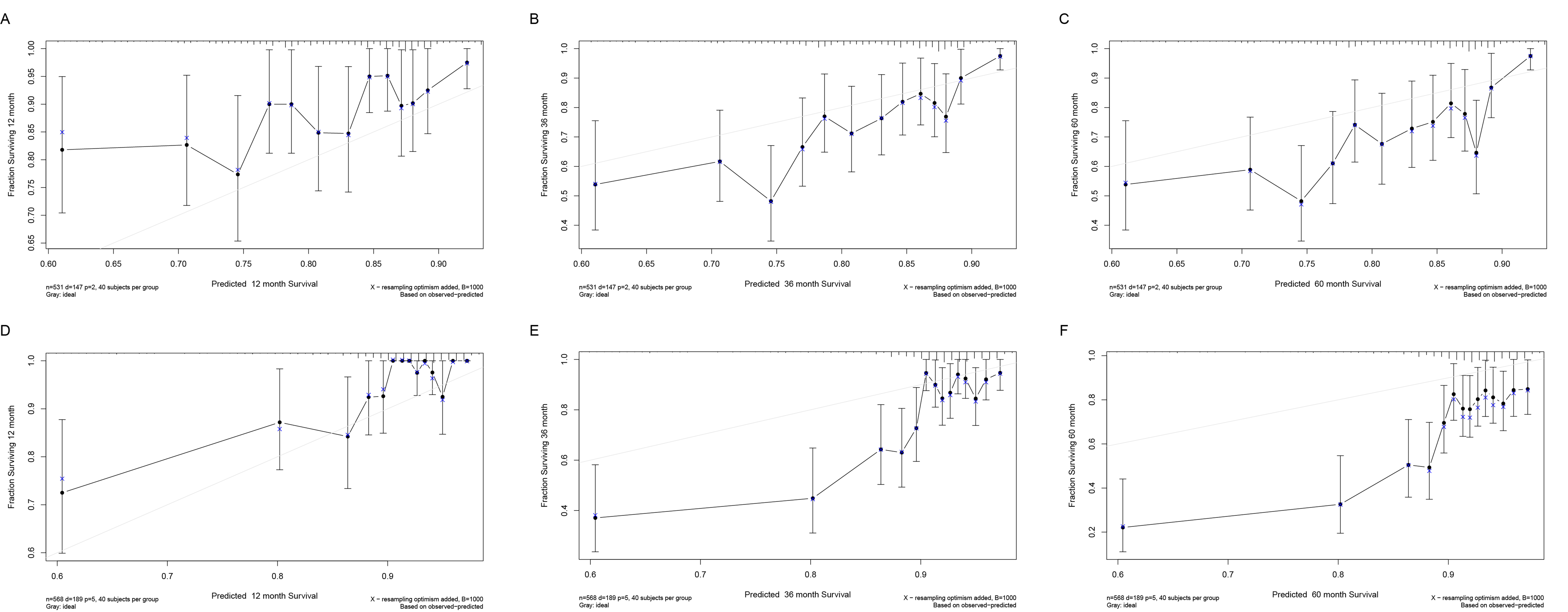

Supplement: Supplementary Figure 8 — Calibration curve for the GSE39582 validation data set. (A–C) Calibration curve of 1-, 3-, and 5-year survival for disease-free surviving patients in GSE39582. (D–F) Calibration curve of 1-, 3-, and 5-year survival for overall surviving patients in GSE39582. [file Image_8.TIF]

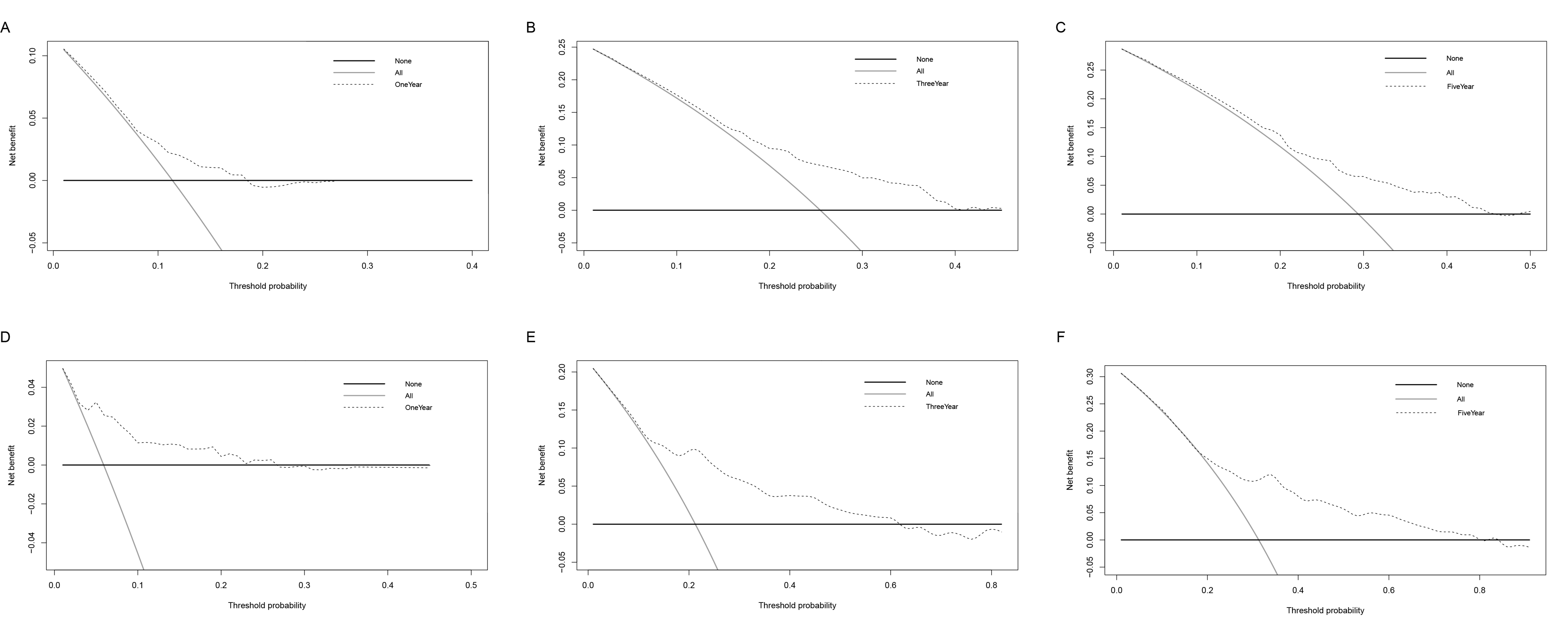

Supplement: Supplementary Figure 9 — DCA for validation of data set GSE39582. (A–C) DCA of 1-, 3- and 5-year survival for disease-free surviving patients in GSE39582. (D–F) DCA of 1-, 3-, and 5-year survival for overall surviving patients in GSE39582. [file Image_9.TIF]

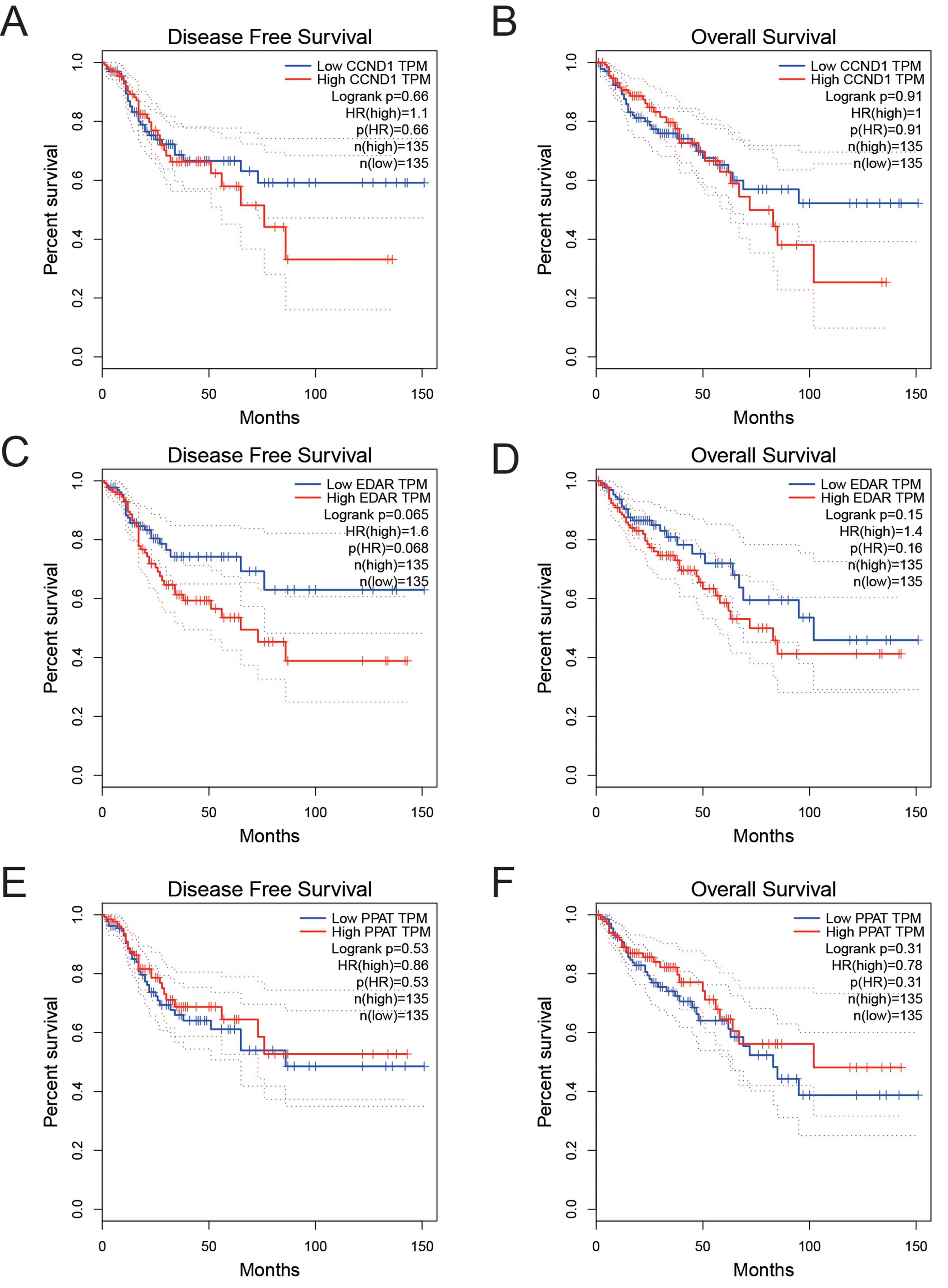

Supplement: Supplementary Figure 10 — Prognostic value of gene expression in GEPIA. (A) CCND1 in DFS. (B) CCND1 in OS. (C) EDAR in DFS. (D) PPAT in OS. (E) PPAT in DFS. (F) PPAT in OS. [file Image_10.TIF]
